# Prevalence and Predictors of Opioid Use Before Spine Surgery: A Multicenter, Cross‐Sectional Observational Study

**DOI:** 10.1002/prp2.70172

**Published:** 2025-09-13

**Authors:** Shania Liu, Jennifer Stevens, Chi Tran, Ashleigh Collins, Jed Duff, Joanna Sutherland, Morgan Oddie, Justine Naylor, Asad Patanwala, Katelyn Jauregui, Jonathan Penm

**Affiliations:** ^1^ Faculty of Medicine and Health, School of Pharmacy The University of Sydney Sydney New South Wales Australia; ^2^ School of Clinical Medicine St Vincent's Clinical Campus, University of New South Wales Sydney New South Wales Australia; ^3^ School of Medicine University of Notre Dame Sydney New South Wales Australia; ^4^ Royal Prince Alfred Hospital Camperdown New South Wales Australia; ^5^ Faculty of Health, School of Nursing Centre for Healthcare Transformation, Queensland University of Technology Brisbane Queensland Australia; ^6^ Rural Clinical School Coffs Harbour Campus, The University of New South Wales Sydney New South Wales Australia; ^7^ St George Hospital Kogarah New South Wales Australia; ^8^ Whitlam Orthopaedic Research Centre Ingham Institute Liverpool New South Wales Australia; ^9^ School of Clinical Medicine UNSW Medicine & Health, South West Sydney Clinical Campus Liverpool New South Wales Australia; ^10^ Pharmacy Department Campbelltown Hospital Campbelltown New South Wales Australia; ^11^ Department of Pharmacy Prince of Wales Hospital Randwick New South Wales Australia

**Keywords:** opioids, preoperative opioid use, spine surgery

## Abstract

Spine surgery is performed on 45 000 Australians annually, and opioids are frequently used for pain management before spine surgery, although concerns about their effectiveness and harms persist. Evidence suggests preoperative opioid use is associated with poorer surgical outcomes, yet the prevalence of opioid use before spine surgery in Australia remains unclear. This study aimed to assess the prevalence and predictors of preoperative opioid use among patients undergoing elective spine surgery across geographical settings in NSW, Australia. A multicenter, cross‐sectional observational study was conducted with patients scheduled for elective spine surgery at four hospitals representing metropolitan, regional, and rural settings from April 2017 to February 2020. Participants completed a pre‐admission questionnaire 2–6 weeks before surgery, providing demographic, clinical, and opioid use data. Preoperative opioid use was reported by 50% (33/66; 95% CI 37%–63%) of participants, with minimal variation across metropolitan (49%), regional metropolitan (54%), and regional hospitals (50%). The most frequently used opioids were oxycodone (32%), codeine (20%), and tapentadol (9%). No significant predictors of opioid use were identified in relation to location, sex, socioeconomic status, smoking, or disability, although trends suggested a higher likelihood of opioid use among patients from lower socioeconomic backgrounds. These findings indicate that 50% of patients use opioids before elective spine surgery, with minimal geographical variation. Given the risks of opioids, efforts to better understand and manage opioid use before spine surgery are warranted. Addressing this issue could lead to more effective pain management strategies and improved patient outcomes.

## 1 Introduction

Approximately 45 000 Australians undergo spine surgery each year [1]. Spine surgery is performed when conservative treatments have failed, when structural abnormalities necessitate surgical intervention, or in the management of back pain related to infection, cancer, or gross instability [2]. Spine surgery also has a role in the management of nerve compression or radiculopathy, which are often associated with neuropathic pain [3]. This subset of patients often experiences more severe symptoms such as radicular pain compared to those with non‐specific low back pain, which may significantly impact their quality of life and functional status.

Opioids are frequently used for pain management prior to spine surgery. International studies report the use of opioids before spine surgery varies widely, with reported rates ranging between 20% and 70% [4, 5, 6]. While opioids are commonly prescribed for preoperative pain, concerns about their effectiveness and the potential for harm are well documented. Evidence suggests that regular opioid use before spine surgery is associated with poorer surgical outcomes, including increased rates of complications, delayed recovery time, and higher levels of postoperative pain [7]. Despite the known risks, there is limited information regarding the prevalence of opioid use prior to spine surgery, particularly in Australia.

A number of factors may influence the prevalence of opioid use before spine surgery in Australia. A 2021 report by the Australian Penington Institute identified significant geographical variation in the prevalence of opioid use, where higher rates of opioid use are reported in regional and rural settings compared with metropolitan settings [8]. Australian data also show a disproportionately high rate of spine surgery performed in the private sector compared to public sector settings [9], possibly reflecting the tendency for public hospitals to manage patients with higher acuity and more complex needs, as well as resource limitations. For example, Harris and Dao [9] reported that publicly performed spinal fusion procedures increased by only 2% over 10 years, compared to a 167% increase in the private sector. It also remains unclear whether factors such as geographical setting and private health insurance coverage influence opioid use before spine surgery. This study aimed to examine the prevalence of any opioid use before elective spine surgery across Australian settings. The secondary objective of the study was to examine potential predictors for opioid use before spine surgery.

## 2 Methods

### 2.1 Study Design

A multicenter, cross‐sectional, observational study was conducted for patients undergoing spine surgery at four hospitals in New South Wales, which consisted of a mix of metropolitan, regional, and rural hospitals [8]. These include:

– A private metropolitan (major city) hospital with 480 beds.
– A public inner regional hospital with 796 beds.
– A private outer regional hospital with 151 beds.
– A public rural hospital with 180 beds.

The study took place from April 2017 to February 2020. This study was approved by the St Vincent's Hospital Human Research Ethics Committee (LNR/17/SVH/155) and conducted in accordance with the Strengthening the Reporting of Observational Studies in Epidemiology (STROBE) guidelines [10].

### 2.2 Study Population

Participants aged 18 years or older, planned for spine surgery, were included in this study. Spine procedures included spine laminectomy, decompression, discectomy, foraminotomy, fusion, and resection. However, individuals assigned for surgery related to cancer, those undergoing a surgical procedure completed under local anesthesia, or those who failed to complete the study questionnaire due to a significant language barrier or medical condition (such as dementia) were excluded. All participants provided written and informed consent before being enrolled in the study.

### 2.3 Data Collection

At the pre‐admission clinic visit, held on average 2–6 weeks prior to spine surgery, participants were asked by study investigators or pre‐admission clinic staff to complete a 26‐item telephone questionnaire. This questionnaire captured clinical and demographic information such as age, sex, Aboriginal or Torres Strait Islander identification, analgesic use, pain severity, residential postcode, highest education level, employment status, and relevant psychological and social background. Participants were not provided with any incentives to take part in this study.

Opioid use was defined as the consumption of opioids by participants for pain relief, at any degree, regardless of frequency, including both regular use and ‘as required’. Daily opioid use was defined as daily opioid use for at least 2 weeks.

Opioids included were those available in Australia at the time of this study: buprenorphine, codeine, fentanyl, hydromorphone, methadone, morphine, oxycodone, tapentadol, and tramadol. Opioids used for opioid use disorder (e.g., methadone oral liquid, sublingual buprenorphine formulations), as well as antitussive opioid formulations were excluded.

Daily oral morphine milligram equivalences (MME) were calculated using established conversion factors [11]. For additional doses taken “as required,” the daily oral MME was calculated by determining the average opioid usage over a 1‐week period and calculating an average daily equivalent quantity.

Non‐opioid analgesics included paracetamol, non‐steroidal anti‐inflammatory drugs (NSAIDs) and gabapentinoids such as gabapentin and pregabalin.

Pain intensity experienced by the participant in the 24 h preceding questionnaire completion was assessed using a validated tool for measuring pain intensity—an 11‐point numerical pain scale rating [12]. A score of zero indicated no pain, while 10 indicated the worst imaginable pain.

Participant residential postcode was used to collect remoteness classification and socioeconomic decile.

Remoteness was determined based on the Australian Statistical Geography Standard‐Remoteness Area (ASGS‐RA) scale [13]. The scale ranged from 1 to 6, with 1 representing major cities, 2 representing inner regional areas, 3 representing outer regional areas, 4 representing remote areas, 5 representing very remote areas, and 6 representing migratory (off‐shore) regions. In addition to the ASGS‐RA classification, we distinguished the capital city setting apart from regional and rural settings per a previous Australian report of opioid‐related harm by the Penington Institute [8].

The socioeconomic decile was derived from the Index of Relative Socio‐economic Disadvantage (IRSD), an index by the Australian Bureau of Statistics. It encompasses various factors such as low educational attainment, low income, high unemployment, and unskilled occupations within postal areas [14]. Decile values ranged from 1 to 10, with decile 1 representing the highest level of disadvantage and decile 10 representing the lowest level of disadvantage.

Psychological and social background data were collected using validated tools, including the two‐item Patient Health Questionnaire (PHQ‐2) to collect depressive traits [15], the two‐item Generalized Anxiety Disorder Questionnaire (GAD‐2) to collect anxiety traits [16] and the five‐item Screener and Opioid Assessment for Patients with Pain (SOAPP‐R version 1.0‐SF) tool to collect data for monitoring aberrant opioid use, including susceptibility to addiction [17], where lower scores indicate less of the symptom/trait measured, and higher scores indicate more of the symptoms/traits measured.

### 2.4 Primary and Secondary Outcomes

The primary study outcome was the prevalence of any opioid use, 2–6 weeks before elective spine surgery across Australian metropolitan, regional, and rural settings. As a secondary outcome, the association between socioeconomic status and daily preoperative opioid use while controlling for covariates was examined.

### 2.5 Sample Size

Based on existing literature [3, 4, 7], we estimated approximately 40% of people awaiting spine surgery are taking opioid analgesics. Using a population survey design with a wide acceptable margin of error of 10% and a 90% confidence interval (CI), a sample size of 65 patients was required. Sample size calculations were performed using the EpiInfo Stat Calc software (Centers for Disease Control and Prevention, Atlanta, Georgia, USA).

### 2.6 Data Analysis

Frequencies were generated for the primary outcome and to compare cohorts who did or did not use opioids before spine surgery.

Multivariable logistic regression was conducted to determine the impact of sex or socioeconomic decile on any opioid use before spine surgery. All statistical analyses were performed using IBM SPSS Statistics Version 27 (IBM, Armonk, NY, USA) and a two‐sided *p* < 0.05 was considered statistically significant.

## 3 Results

In total, 66 participants were recruited (Table 1). The mean age was 55.4 (standard deviation [SD] 13.7) years and 21 (31.8%) were female. Median remoteness classification was 1 (interquartile range [IQR] 1) and median socioeconomic decile was 4 (IQR 6). Among the total cohort, 29 (43.9%) participants reported any non‐opioid analgesic use and 23 (34.8%) reported daily opioid analgesic use. There were 33 (50%) patients who reported any opioid analgesic use during the past 2 weeks (95% CI 37%–63%). Rates of any preoperative opioid use among participants admitted to the metropolitan hospital were 48.6% (18/37), 54.2% (13/24) among those admitted to the inner regional hospital, and 50% (2/4) among participants admitted to the outer regional hospital (Table A1). The most frequently used opioids were oxycodone (31.8%, 21/66), codeine (19.7%, 13/66) and tapentadol (9.1%, 6/66). Overall median MME per day was 30 (IQR 52.35; Table 1).

**TABLE 1:** Characteristics of patients awaiting elective spine surgery.

Demographics	*N* (*n* = 66)
Age; years	55.4 (13.7)
Sex; female	21 (31.8%)
Hospital Site	
Metropolitan hospital	37 (56.1%)
Inner regional hospital	24 (36.4%)
Outer regional hospital	4 (6.1%)
Rural hospital	1 (1.5%)
Remoteness (ASGS‐RA)	
1	43 (65.2%)
2	18 (27.3%)
3	5 (7.6%)
Socioeconomic decile (IRSD)	
1	7 (10.6%)
2	8 (12.1%)
3	6 (9.1%)
4	12 (18.2%)
5	5 (7.6%)
6	3 (4.5%)
7	3 (4.5%)
8	2 (3%)
9	6 (9.1%)
10	14 (21.2%)
Identification as Aboriginal or Torres Strait Islander	
Aboriginal	2 (3%)
Torres Strait Islander	0 (0%)
Pain experience	
History of chronic pain	47 (71.2%)
Pain score in last 24 h^a^	6 (2.9)
Percentage relief from pain medications	36.7 (27.7)
Pain experienced on most days	54 (81.8%)
Pain experienced in two or more sites	41 (62.1%)
Preoperative analgesia use	
Non‐opioid analgesia use	29 (43.9%)
Daily opioid analgesia use	23 (34.8%)
Any opioid analgesia use	33 (50%)
Opioid use by type	
Oxycodone	21 (31.8%)
Codeine	13 (19.7%)
Tapentadol	6 (9.1%)
Tramadol	4 (6.1%)
Morphine	2 (3%)
Fentanyl	1 (1.5%)
Total morphine milligram equivalents	30 (52.35 [3–500])
Highest education level	
Grade 10 or below	22 (33.3%)
Grade 12	8 (12.1%)
University or TAFE	35 (53%)
Employment status	
Unemployed	6 (9.1%)
Employed	26 (39.4%)
Retired	19 (28.8%)
Student	1 (1.5%)
Unable to work due to disability	10 (15.2%)
Other	3 (4.5%)
Psychological background	
Feeling nervous, anxious or on edge^b^	2 (1)
Worrying^b^	1.7 (1.1)
Feeling little interest or pleasure^b^	1.9 (1.1)
Feeling down, depressed or hopeless^b^	1.6 (1)
Experiencing mood swings^c^	2.4 (1)
Other relevant social or medication history^c^	
Smoking a cigarette within an hour of waking up	1.4 (1.1)
Medications taken other than prescribed	1.4 (0.9)
Recreational drug use in past 5 years	1.3 (0.8)
Legal problems or arrested	1.2 (0.5)
Likelihood of developing addiction to pain medication	1.2 (0.6)

*Note:* Values are expressed as mean (SD), median (IQR), or number (proportion).

Abbreviations: ASGS‐RA, Australian Statistical Geography Standard‐Remoteness Area; IRSD, Index of Relative Socio‐Economic Disadvantage; IQR, interquartile range; SD, standard deviation; TAFE, technical and further education.

^a^ Using 11‐point numerical pain rating scale (0 = no pain, 10 = worst imaginable pain).

^b^ Using 5‐point Likert scale (0 = not at all, 4 = nearly every day). The Generalized Anxiety Disorder 2‐item (GAD‐2) screening tool was used.

^c^ Using 6‐point Likert scale (0 = never, 5 = very often). The Patient Health Questionnaire‐9 (PHQ‐9) was used.

Univariate differences between opioid naïve patients and those using any opioids before elective spine surgery are reported in Table 2. Participants using any opioids before surgery were more likely to report concurrent non‐opioid analgesic use (63.6%, 21/33 vs. 24.2%, 8/33) and reported a higher mean percentage pain relief from analgesics (46.4 [SD 24.7] vs. 27 [SD 27.4]) compared to those not using opioids before surgery. Among participants using any preoperative opioids, a greater proportion reported inability to work due to disability (24.2%, 8/33 vs. 6.1%, 2/33) and tobacco use (mean [SD], 1.7 [1.4] vs. 1.1 [0.5]) compared to opioid naïve participants (Table A2).

**TABLE 2:** Characteristics of patients awaiting elective spine surgery.

Demographics	Not using opioids preoperatively (*n* = 33)	Using opioids preoperatively (*n* = 33)
Age; years	57.5 (13.5)	53.3 (13.8)
Sex; female	10 (30.3%)	11 (33.3%)
Hospital site		
Metropolitan hospital	19 (57.6%)	18 (54.5%)
Inner regional hospital	11 (33.3%)	13 (39.4%)
Outer regional hospital	2 (6.1%)	2 (6.1%)
Rural hospital	1 (3%)	0 (0%)
Public or private hospital		
Public	21 (63.6%)	20 (60.6%)
Private	12 (36.4%)	13 (39.4%)
Remoteness (ASGS‐RA)	1 (0.5 [1–3])	1 (1 [1–3])
1	25 (75.8%)	18 (54.5%)
2	7 (21.2%)	11 (33.3%)
3	1 (3%)	4 (12.1%)
Socioeconomic decile (IRSD)	5 (6.5 [1–10])	4 (6 [1–10])
1	4 (12.1%)	3 (9.1%)
2	4 (12.1%)	4 (12.1%)
3	0 (0%)	6 (18.2%)
4	7 (21.2%)	5 (15.2%)
5	2 (6.1%)	3 (9.1%)
6	1 (3%)	2 (6.1%)
7	2 (6.1%)	1 (3%)
8	2 (6.1%)	0 (0%)
9	3 (9.1%)	3 (9.1%)
10	8 (24.2%)	6 (18.2%)
Identification as Aboriginal or Torres Strait Islander		
Aboriginal	1 (3%)	1 (3%)
Torres Strait Islander	0 (0%)	0 (0%)
Preoperative analgesia use		
Non‐opioid analgesia use	8 (24.2%)	21 (63.6%)
Pain experience		
History of chronic pain	22 (66.7%)	25 (75.8%)
Pain score in last 24 h^a^	5.4 (3.2)	6.7 (2)
Percentage relief from pain medications	27 (27.4)	46.4 (24.7)
Pain experienced on most days	24 (72.7%)	30 (90.9%)
Pain experienced at surgery site	24 (72.7%)	25 (75.8%)
Pain experienced in two or more sites	17 (51.5%)	24 (72.7%)
Highest education level		
Grade 10 or below	10 (30.3%)	12 (36.4%)
Grade 12	5 (15.2%)	3 (9.1%)
University or TAFE	17 (51.5%)	18 (54.5%)
Employment status		
Unemployed	3 (9.1%)	3 (9.1%)
Employed	15 (45.5%)	11 (33.3%)
Retired	9 (27.3%)	10 (30.3%)
Student	0 (0%)	1 (3%)
Unable to work due to disability	2 (6.1%)	8 (24.2%)
Other	3 (9.1%)	0 (0%)
Psychological background		
Feeling nervous, anxious or on edge^b^	1.8 (1)	2.1 (1.1)
Worrying^b^	1.6 (1.1)	1.7 (1.1)
Feeling little interest or pleasure^b^	1.8 (1.1)	2 (1.1)
Feeling down, depressed or hopeless^b^	1.5 (0.9)	1.7 (1.1)
Experiencing mood swings^c^	2.1 (1)	2.6 (1.1)
Other^c^		
Smoking a cigarette within an hour of waking up	1.1 (0.5)	1.7 (1.4)
Medications taken other than prescribed	1.5 (1.1)	1.3 (0.5)
Recreational drug use in past 5 years	1.4 (0.9)	1.3 (0.8)
Legal problems or arrested	1.1 (0.4)	1.2 (0.6)
Likelihood of developing addiction to pain medication	1.1 (0.3)	1.3 (0.8)

*Note:* Values are expressed as mean (SD), median (IQR [range]), or number (proportion).

Abbreviations: ASGS‐RA, Australian Statistical Geography Standard‐Remoteness Area; IRSD, Index of Relative Socio‐Economic Disadvantage; IQR, interquartile range; SD, standard deviation; TAFE, technical and further education.

^a^ Using 11‐point numerical pain rating scale (0 = no pain, 10 = worst imaginable pain).

^b^ Using 5‐point Likert scale (0 = not at all, 4 = nearly every day). The Generalized Anxiety Disorder 2‐item (GAD‐2) screening tool was used.

^c^ Using a 6‐point Likert scale (0 = never, 5 = very often). The Patient Health Questionnaire‐9 (PHQ‐9) was used.

Multivariable regression showed no association between sex or socioeconomic background and opioid use prior to spine surgery (Wald test *p* > 0.05; Table 3). The Hosmer–Lemeshow test indicated a good fit of the data to the logistic regression model (*p* > 0.05).

**TABLE 3:** Multivariable logistic regression analyses for predictors and covariates of preoperative opioid use before elective spine surgery.

	*N* ^a^ (*n* = 66)
Variable	Adjusted OR (95% CI)
Sex	
Male	[Reference]
Female	1.16 (0.41–3.29)
Socio‐economic decile (IRSD)	
Greater than 3	[Reference]
3 or less	1.65 (0.62–4.42)

Abbreviations: CI, confidence interval; IRSD, Index of Relative Socio‐Economic Disadvantage; OR, odds ratio.

^a^ Hosmer–emeshow goodness of fit (*p* = 0.511).

## 4 Discussion

Our study found that 50% of patients used opioids at 2 to 6 weeks before elective spine surgery (33/66; 95% CI 37% to 63%). This is consistent with rates reported in other studies, with opioid use ranging between 20% and 70% prior to spine surgery [4, 5, 7]. Preoperative opioid use is a significant predictor of long‐term opioid use after surgery and has been linked with poorer surgical outcomes. Specifically, it has been linked with decreased quality of life and reduced cost‐effectiveness of treatment [18], as well as an increased risk of complications, readmissions, and higher overall costs [19]. Additionally, a systematic review conducted by Yernini et al. highlights the negative impacts of preoperative opioid use on clinical outcomes across various spine surgeries [7]. Similarly, a retrospective review by Kha et al. underscores these concerns, particularly regarding outcomes in lumbar spine surgery where patients on chronic opioid therapy prior to surgery are associated with longer hospital stays and prolonged opioid use after surgery compared to patients not on chronic opioid therapy (211 days vs. 79 days; *p* = 0.001) [20]. These findings emphasize the need for targeted strategies to manage preoperative opioid use, which is crucial for improving surgical outcomes and patient well‐being in spine surgery, enhancing postsurgery recovery and achieving better long‐term results. In certain clinical situations, such as acute vertebral compression fractures, short‐term opioid use is clinically indicated in accordance with clinical guidelines to manage severe pain and facilitate early mobilization. Australian clinical guidelines support this approach, recognizing that opioid use in these acute pain settings is justified when the benefits outweigh the risks. They also emphasize the importance of patient monitoring and minimizing both the duration and dose of opioid therapy [21, 22]. This balanced strategy promotes personalized pain management that optimizes effectiveness while minimizing potential harm.

This study found that factors such as location, sex, socioeconomic status, smoking, and disability did not predict preoperative opioid use among patients undergoing elective spine surgery. In contrast, Liu et al. identified socioeconomic status, alcohol consumption, and tobacco use as significant predictors of long‐term opioid use following orthopedic procedures (including hip, knee, and shoulder arthroplasty), which may reflect different predictor profiles across different surgical populations [23]. This difference could suggest that spine surgery patients face unique challenges or fewer options in pain management compared to those undergoing orthopedic procedures. Further research with larger sample sizes and more comprehensive assessments may provide additional insights into the contributors to preoperative opioid use in elective spine surgery patients. It may also help better understand the factors influencing preoperative opioid use across different hospital geographical settings.

Our study has several limitations. Firstly, the use of patient self‐reported data introduces the possibility of response and recall biases, raising concerns about the accuracy of reported opioid use. Additionally, the prevalence of opioid dependency prior to surgery remains uncertain, as we did not assess dependency. Analgesic usage trends may have varied over the 2–6 weeks preceding surgery, potentially impacting reported rates of preoperative opioid use. We did not collect data on other comorbid conditions beyond chronic pain, limiting our understanding of the overall comorbidity burden among participants. Data on non‐opioid analgesics, including non‐steroidal anti‐inflammatory drugs (NSAIDs), were also not collected in this study. Therefore, their role in preoperative pain management and any influence on opioid use remain unclear. This limitation highlights an important area for future research.

Our study did not collect clinical or radiographic measures of disease severity. Therefore, we are unable to determine if the lower opioid use seen in private sector patients reflects differences in spinal disease severity. Although Australian data indicate that public hospitals typically manage patients with higher acuity and more complex needs [9], the absence of disease severity data in our study limits the ability to assess this as a potential factor influencing opioid prescribing. Additionally, poorer outcomes observed in patients using opioids preoperatively may be related to more severe underlying disease rather than opioid use itself. As our study lacks measures of disease severity, confounding related to disease severity cannot be excluded. These gaps identify key priorities for future studies.

The absence of specific indications for opioid use may potentially lead to an overrepresentation of preoperative opioid use in our cohort. Additionally, the remoteness classifications used in our study were not sensitive enough to capture variations in opioid use rates between metropolitan and regional locations. As our study was conducted within one state in Australia, the generalizability of our findings to different Australian states and territories may be limited.

In conclusion, our study found half of spine surgery patients were taking opioids before spine surgery, with no significant differences observed between metropolitan and regional or rural settings. While regional variations were not significant, further research could enhance our understanding of factors influencing preoperative opioid use. Addressing this issue remains crucial for improving patient outcomes across healthcare settings.

## Author Contributions

Concept and design: J.S., A.P., J.N., J.P. Acquisition, analysis, or interpretation of data: S.L., A.C., J.D., J.S., M.O., J.P. Drafting of the manuscript: C.T., S.L. Critical revision of the manuscript for important intellectual content: S.L., C.T., K.J. Statistical analysis: S.L. Supervision: J.S., J.N., A.P., J.P. Review of the manuscript: All authors.

## Conflicts of Interest

The authors declare no conflicts of interest.

## Data Availability

The data that support the findings of this study are available on request from the corresponding author. The data are not publicly available due to privacy or ethical restrictions.

## Appendix A

**TABLE A1:** Rates and quantities of any preoperative opioid use by hospital site prior to elective spine surgery.

Hospital site	Any preoperative opioid use, *n* (%)	Morphine milligram equivalents, median (IQR)
Metropolitan hospital	18/37 (49%)	25 (62.3)
Inner regional hospital	13/24 (54%)	30 (53.9]
Outer regional hospital	2/4 (50%)	18.8 (42.3)

Abbreviation: IQR, interquartile range.

**TABLE A2:** Rates and quantities of daily preoperative opioid use by hospital site prior to elective spine surgery.

Hospital site	Daily preoperative opioid use, *n* (%)	Morphine milligram equivalents, median (IQR)
Metropolitan hospital	11/37 (30%)	45 (55.9)
Inner regional hospital	10/24 (42%)	38 (60.9)
Outer regional hospital	2/4 (50%)	19 (11.3)

Abbreviation: IQR, interquartile range.
